# Therapeutic and Contrast Agents for Photoacoustic Imaging-Guided Photothermal Therapy: A Narrative Review

**DOI:** 10.7150/ntno.96286

**Published:** 2024-08-01

**Authors:** Donni Kis Apriyanto, Mitrayana, Andreas Setiawan, Rini Widyaningrum

**Affiliations:** 1 Department of Physics, Faculty of Mathematics and Natural Sciences, Universitas Gadjah Mada, Yogyakarta 55281, Indonesia.; 2 Department of Physics, Faculty of Mathematics and Natural Sciences, Universitas Lampung, Bandar Lampung 35141, Indonesia.; 3 Department of Physics, Faculty of Science and Mathematics, Universitas Kristen Satya Wacana, Salatiga 50711, Indonesia.; 4 Department of Dentomaxillofacial Radiology, Faculty of Dentistry, Universitas Gadjah Mada, Yogyakarta 55281, Indonesia.

**Keywords:** near-infrared wavelength, radiation, absorption, *in vitro*, * in vivo*

## Abstract

Photoacoustic imaging is a hybrid modality that combines high-contrast and spectroscopy-based optical imaging specificity with the high spatial resolution of ultrasonography. This review highlights the development and progress of photoacoustic imaging technology over the past decade. This imaging technology has evolved to be more user-friendly, cost-effective, and portable, demonstrating its potential for diverse clinical applications. A potential clinical application lies in the use of photoacoustic imaging as a guiding tool for photothermal therapy. This review was conducted by initially filtering through three databases, namely, Google Scholar, PubMed, and Scopus, resulting in 460 articles published between 2019 and May 2023. Of these, 54 articles were deemed suitable for review after identification. The selected articles were research papers focusing on the development of therapeutic agents that enhance contrast in photoacoustic imaging. All reviewed articles tested these agents both *in vitro* and *in vivo*. This review focuses on wavelength absorption and radiation sources for photothermal therapy. The developed agents predominantly used NIR-I wavelengths, whereas the NIR-II region has been less explored, indicating significant potential for future research. This review provides comprehensive insights into the advancement of compounds serving as therapeutic agents and contrast agents in photoacoustic imaging-guided photothermal therapy.

## Introduction

Photoacoustic imaging is a hybrid modality that combines high-contrast and spectroscopy-based optical imaging specificity with the high spatial resolution of ultrasonography 1. Several instrument configurations in photoacoustic imaging are employed: optical resolution-photoacoustic microscopy, acoustic resolution-photoacoustic microscopy, photoacoustic tomography/computed tomography, and photoacoustic endoscopy 2. Each configuration has different resolutions and depths and can be used as required. A review of the development and progress of photoacoustic imaging technology over the past decade revealed that this imaging technology has evolved to be more user-friendly, cost-effective, and portable, demonstrating its potential for diverse clinical applications 3. Research involving the application of photoacoustic imaging techniques as monitoring and detection tools in the medical field has been extensive. These include monitoring blood clots during microbubble-assisted sonothrombolysis 4, studying hemodynamic changes caused by blood pressure variations 5, assessing the efficacy of the vascular disrupting agent 5,6-dimethylxanthenone-4-acetic acid through quantitative analysis 6, and studying deep vascularization for regenerative applications 7. Furthermore, photoacoustic imaging holds potential as a guiding tool for various medical procedures requiring intervention, such as drug delivery, therapy, surgery, and biopsy 8.

In addition to diagnostic applications, photoacoustic research extensively focuses on the development of diagnostic and therapeutic systems (theranostics) and drug monitoring strategies 9. Photothermal therapy (PTT) is currently under development and uses photoacoustic imaging as a guiding tool. In addition to conventional cancer therapies, PTT represents an alternative and innovative approach to cancer treatment. PTT is a therapeutic modality that uses light-absorbing agents to heat and selectively destroy cancer cells or other abnormal tissues without harming healthy cells 10. The hybridization of photoacoustic imaging and PTT demonstrates clear advantages in monitoring and treating tumors because of its high efficiency, spatial specificity, fewer side effects, and ease of operation 11.

Photoacoustic imaging has been extensively investigated as a guide for PTT. These studies have focused on various compounds that assist in disease therapy and agents to enhance the contrast of photoacoustic imaging results. These agents assist in the more precise visualization of targets such as tumors or other diseases, especially at the cellular level. Using photoacoustic imaging, medical professionals can obtain valuable information about tumors' location, size, and characteristics. In addition to their use in diagnosis, these agents possess therapeutic properties. They can generate heat when exposed to light radiation, which can be utilized to destroy or damage cancer cells in photothermal therapy. This approach allows for more targeted and precise treatment, minimizing damage to surrounding healthy tissue. Compounds used as agents in PTT are formulated using various compound mixtures; as a result, they possess different maximum absorption wavelengths. Therefore, this review summarizes recent advancements in agents for photoacoustic imaging as guides for PTT based on their components, absorption wavelength ranges, and *in vitro* and *in vivo* PTT applications.

Furthermore, this review can serve as a reference for the development of agents that enhance image contrast in photoacoustic imaging and possess therapeutic properties for treating diseases such as cancer. Thus, these potential agents can be highly efficient and effective diagnostic and therapeutic tools. This review also provides conclusions and a brief discussion on the challenges and perspectives and the potential future directions of PTT agents that simultaneously enhance photoacoustic imaging contrast.

## Methods

**Figure 1:**
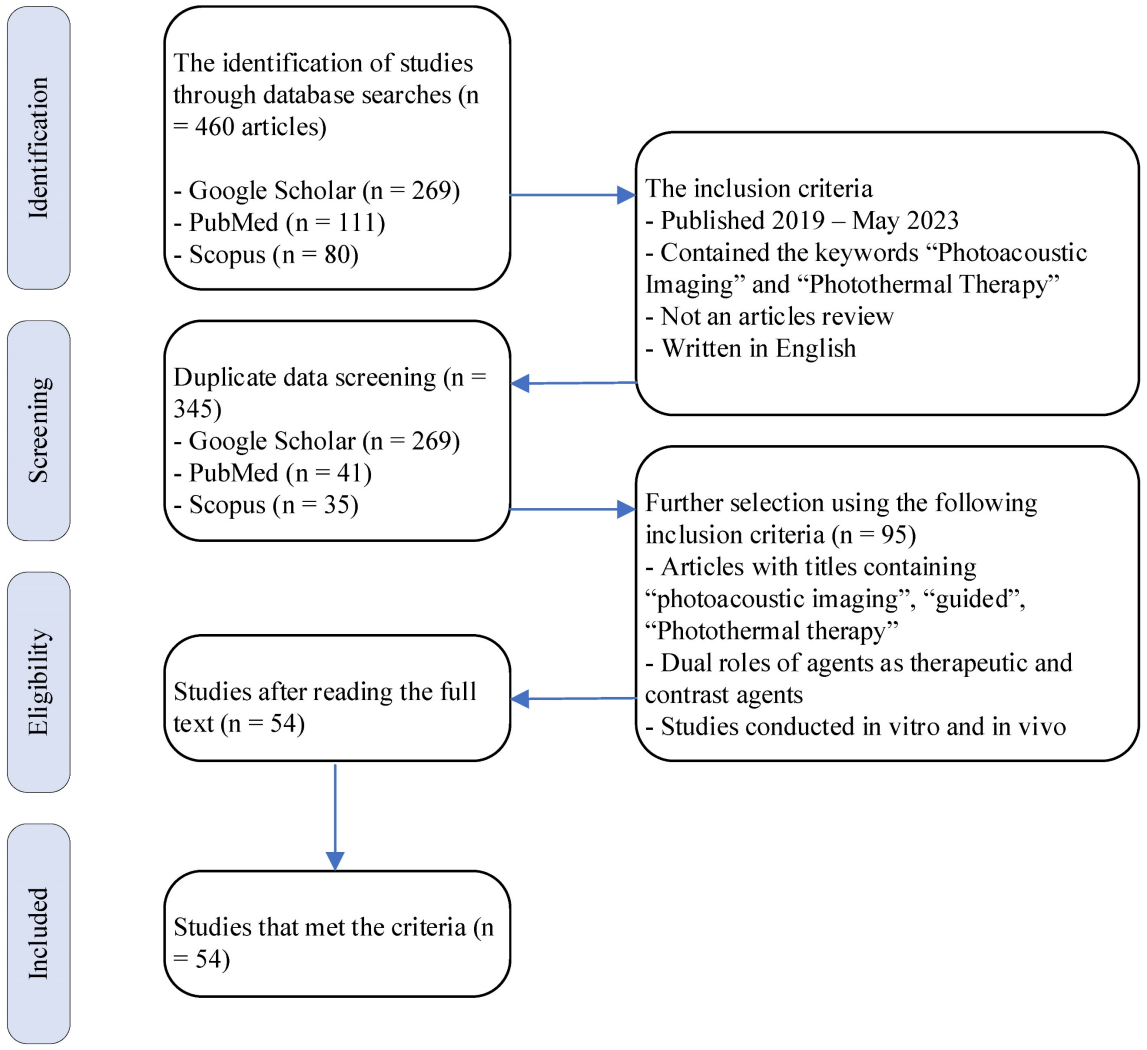
Selection diagram and literature search.

A comprehensive literature search was conducted to filter articles related to photoacoustic imaging-guided PTT. Articles were obtained from the Google Scholar, PubMed, and Scopus databases. The literature search used the keywords “photoacoustic imaging” and “photothermal therapy.” The Boolean operator “AND” and phrase searching (“ ”) were explicitly used to facilitate the search. Clinical trials, case series, case reports, and other relevant articles were considered for review. The selected articles were those published between early 2019 and May 2023. The identified review articles should have been included in the initial evaluation. The initial assessment involved identifying titles through literature searches. Articles not written in English or inaccessible in full text were excluded from the initial evaluation. A flowchart of the search and literature selection process is shown in **Figure 1**. The included studies were evaluated and then organized in a table based on the article title, agent and compound type, cells used *in vitro* or *in vivo*, peak absorption wavelength, photoacoustic wavelength, PTT laser wavelength, and compound temperature change when exposed to laser irradiation.

The exclusion criteria were applied after creating the table to eliminate articles. Further selection of articles was based on titles containing “photoacoustic imaging,” “guided,” and “photothermal therapy.” In addition, articles featuring compounds used as therapeutic and contrast agents were considered. Articles combining *in vitro* and *in vivo* methods were selected for inclusion in this literature review.

## Results and Discussion

Initial screening across the three databases yielded 460 articles. After removing duplicates, 345 articles were included in the initial evaluation. Based on further selection using the following inclusion criteria, 95 articles were selected for further review. During the subsequent review process, 54 articles were deemed suitable for review (**Figure 1**). The summary of the article review is organized into **Table 1**, **Table 2**, **Table 3**, and **Table 4**. **Table 1** presents the optical characteristics of the agent compounds, including the agent name, type, absorption wavelength (λ_abs_), wavelength used for generating photoacoustic images (λ_PA_), imaging modality used, and tool used for image generation. **Table 2** summarizes the distribution of agents based on their absorption wavelengths. **Table 3** summarizes the thermal characteristics of the agent compounds in the *in vitro* methods. This includes the agent's name, cells used *in vitro*, therapeutic technique employed, radiation wavelength delivered (λ_Rad_), radiation source power, duration of agent radiation, temperature increase, and temperature achieved during the provided radiation time, and photothermal conversion efficiency of the agent. **Table 4** presents data related to the thermal characteristics of the agent compounds in the *in vivo* methods. It contains the agent's name, cells implanted in experimental animals, therapeutic technique used, tool for visualizing the agent's temperature in experimental animals, radiation wavelength delivered (λ_Rad_), radiation source power, duration of agent radiation, temperature increase, and temperature achieved during the provided radiation time.

### Optical characteristics of the agents

This review categorizes agents into organic, inorganic, hybrid organic-inorganic, organic dye, organic-inorganic dye, and dye categories. Organic or inorganic refers to compounds formed on basis of the organic or inorganic components, whereas hybrid organic-inorganic refers to mixed compounds formed from organic and inorganic components. Organic and organic-inorganic dyes are colorants derived from organic components or a mixture of organic-inorganic components. This classification is based on the information in the articles or material component of the agents used in the studies reviewed in this work.

**Figure 2:**
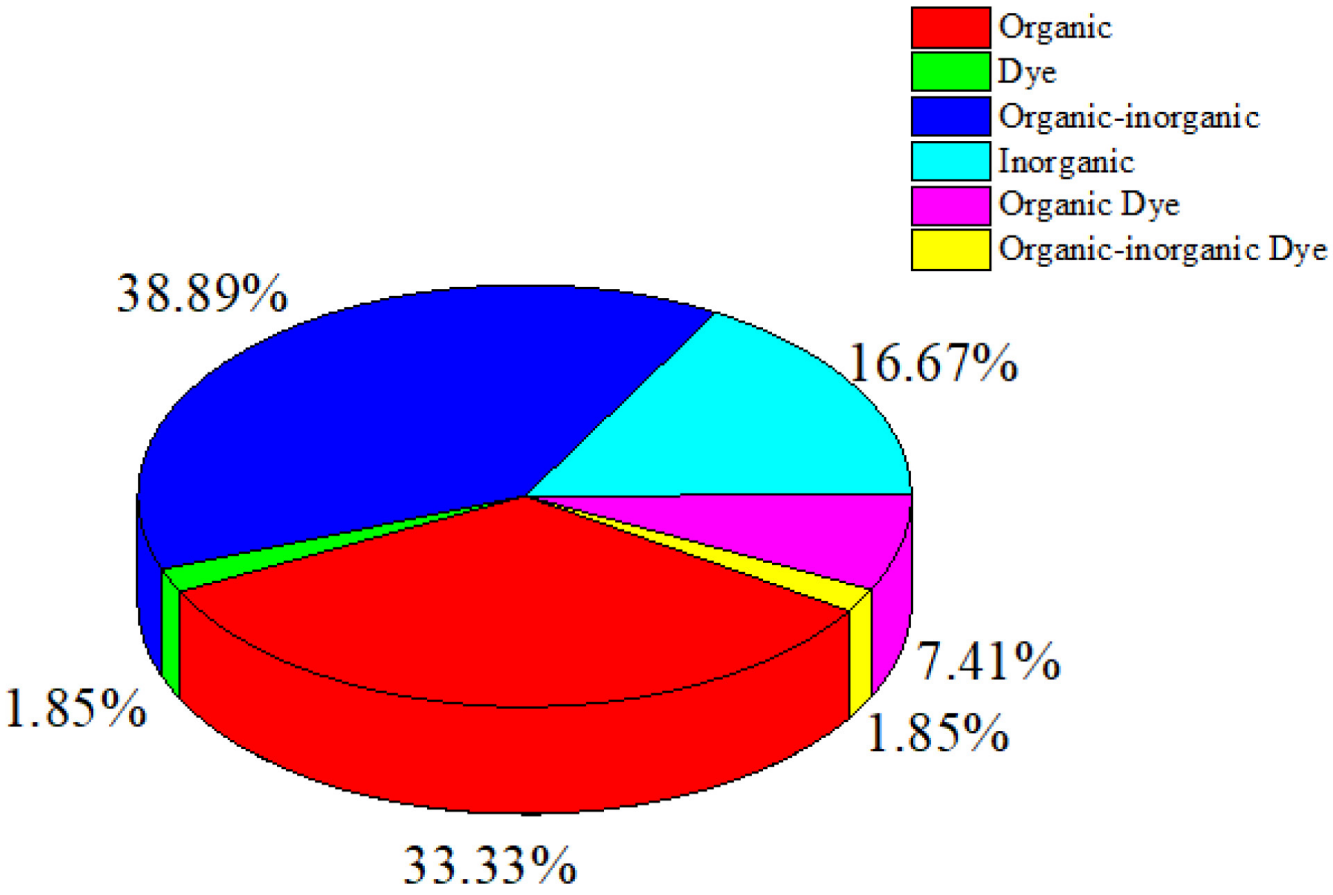
Distribution of contrast agent types, including organic, inorganic, hybrid organic-inorganic, organic dye, organic-inorganic dye, and dye.

**Figure 2** indicates that the compounds used in photoacoustic imaging-guided PTT are mainly derived from a combination of organic-inorganic components, with compounds originating from organic and inorganic elements following closely. The prevalent use of organic materials stems from their potential for biocompatibility and biodegradability within biological systems compared with inorganic materials. Organic materials also exhibit high absorption in the far-red or near-infrared (NIR) region, presenting significant potential for PTT 12. As shown in **Table 1** and **Figure 2**, studies employing dyes as contrast and therapeutic agents are fewer than those employing other agent types.

One of the main challenges in photoacoustic imaging is the limited depth of light penetration in biological tissues, especially in the visible and NIR-I regions. Additionally, some non-contact photoacoustic imaging methods may encounter constraints related to signal travel distance, especially when imaging deep tissues. These constraints can lead to lower resolution in deep tissues than direct contact imaging methods (Ying et al., 2024). These limitations reduce the effectiveness of imaging deep tissues and organs, limiting their clinical applications. Therefore, to enhance treatment effectiveness, developing techniques capable of overcoming these barriers is necessary, such as using radiation sources with longer wavelengths or developing contrast agents with optimized optical properties for better tissue penetration.

The wavelength spectrum regions of NIR can be divided into three channels: NIR-I (700-900 nm), NIR-IIa (1300-1400 nm), and NIR-IIb (1500-1700 nm) 13. In this review, they were categorized into the visible region (400-700 nm), NIR-I (700-1000 nm), and NIR-II (1000-1700 nm) 14 because of the presence of agents with absorption wavelengths in the visible light region.

**Figure 3:**
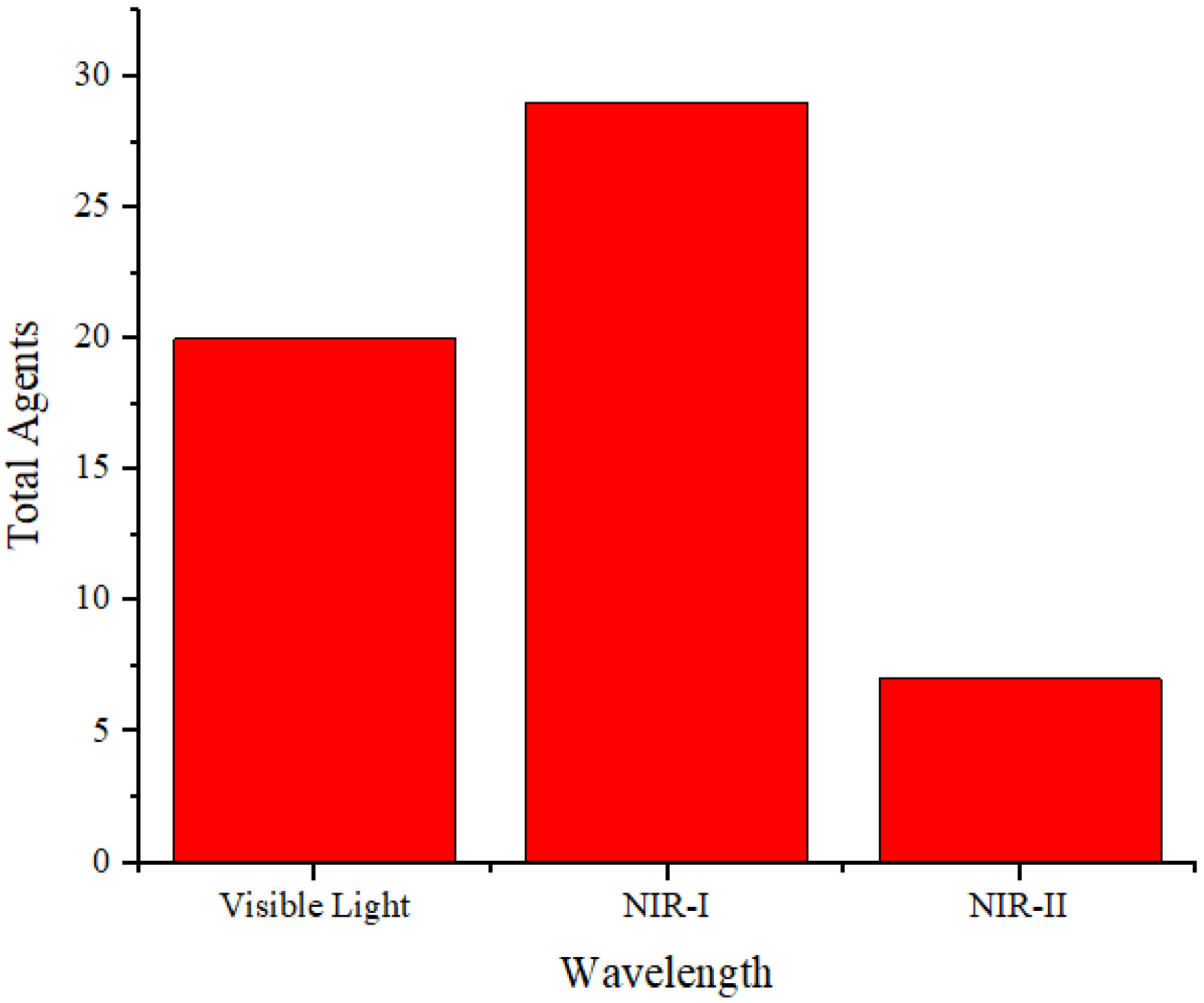
Distribution of the wavelength absorbance of the agents.

**Table 2** summarizes the distribution of agents based on their absorption wavelengths, which is illustrated in **Figure 3**. In **Figure 3** presents the development of agents that absorb at specific wavelengths. More agents are designed to be absorbed in the NIR-I wavelength region. However, studies using NIR-II are still fewer than those utilizing other wavelength ranges. The use of the NIR-II window (1000-1700 nm) was limited because of the absence of contrast agents with absorption capabilities in the NIR-II region 69. They reported synthesizing photoacoustic contrast agents that could be absorbed in both the NIR-I (750 nm) and NIR-II (1064 nm) regions, directly comparing the resulting photoacoustic images. The findings indicated that the signal-to-noise ratio of the photoacoustic images generated at a wavelength of 1064 nm was higher than that at 750 nm.

**Figure 4:**
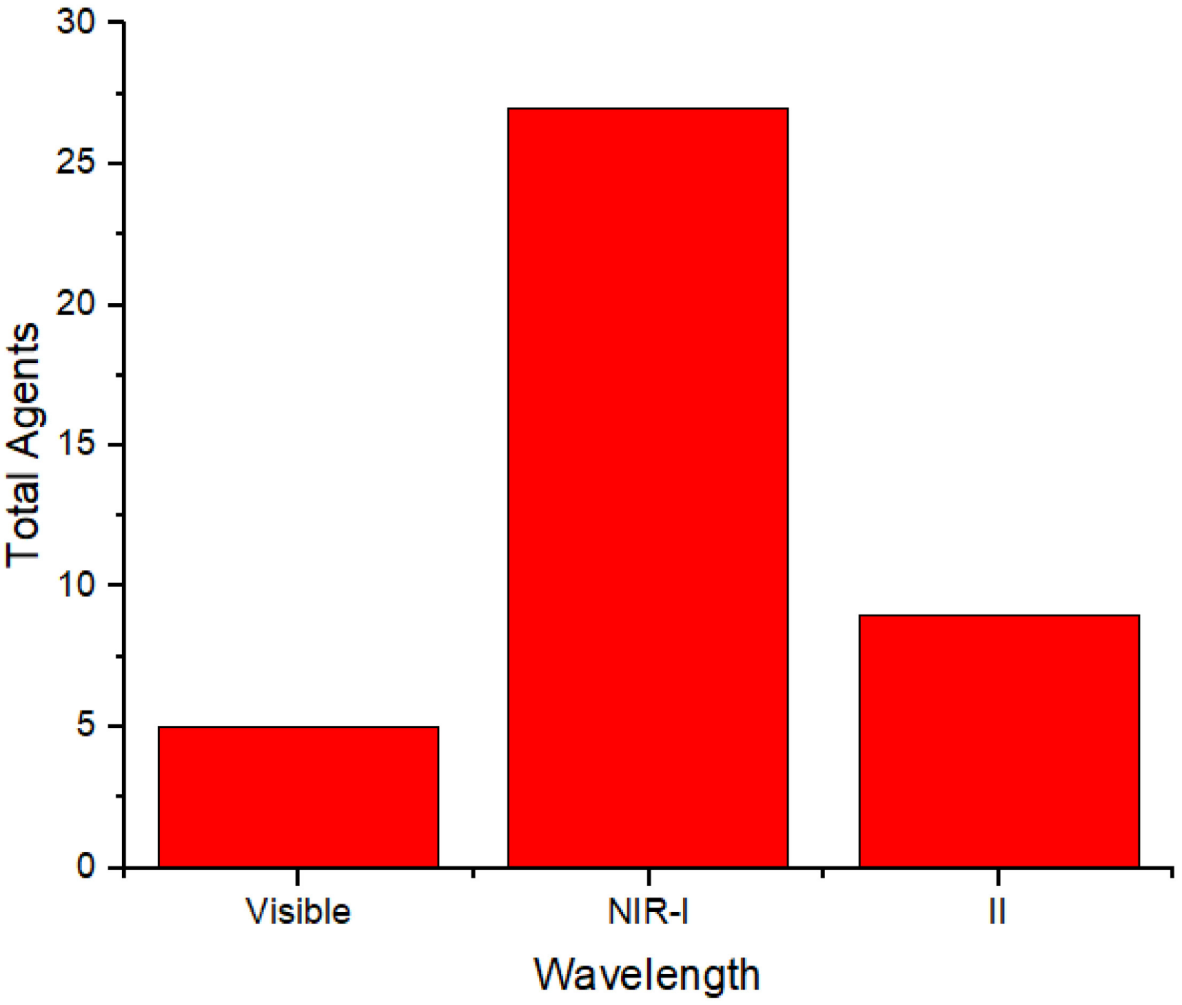
Distribution of photoacoustic imaging wavelengths.

**Figure 4** displays the distribution of wavelength usage as a radiation source for generating photoacoustic images. In this figure, the NIR-I region is more extensively used as a radiation source. The number of agents in each absorption region in **Figure 3** is mostly the same as that in each wavelength region used to generate photoacoustic images in **Figure 4**. This indicates that the selection of wavelengths as a source for photoacoustic imaging is adjusted according to the agents' absorption wavelengths to maximize the obtained images. Moreover, the choice of photoacoustic imaging wavelengths is also determined by the depth of the object being imaged.

### PTT using in vitro methods

**Table 3** summarizes the thermal characteristics of the agent compounds in the *in vitro* methods, including the cells used *in vitro*, laser irradiation, agent temperature increase, and agent photothermal conversion efficiency.

**Figure 5:**
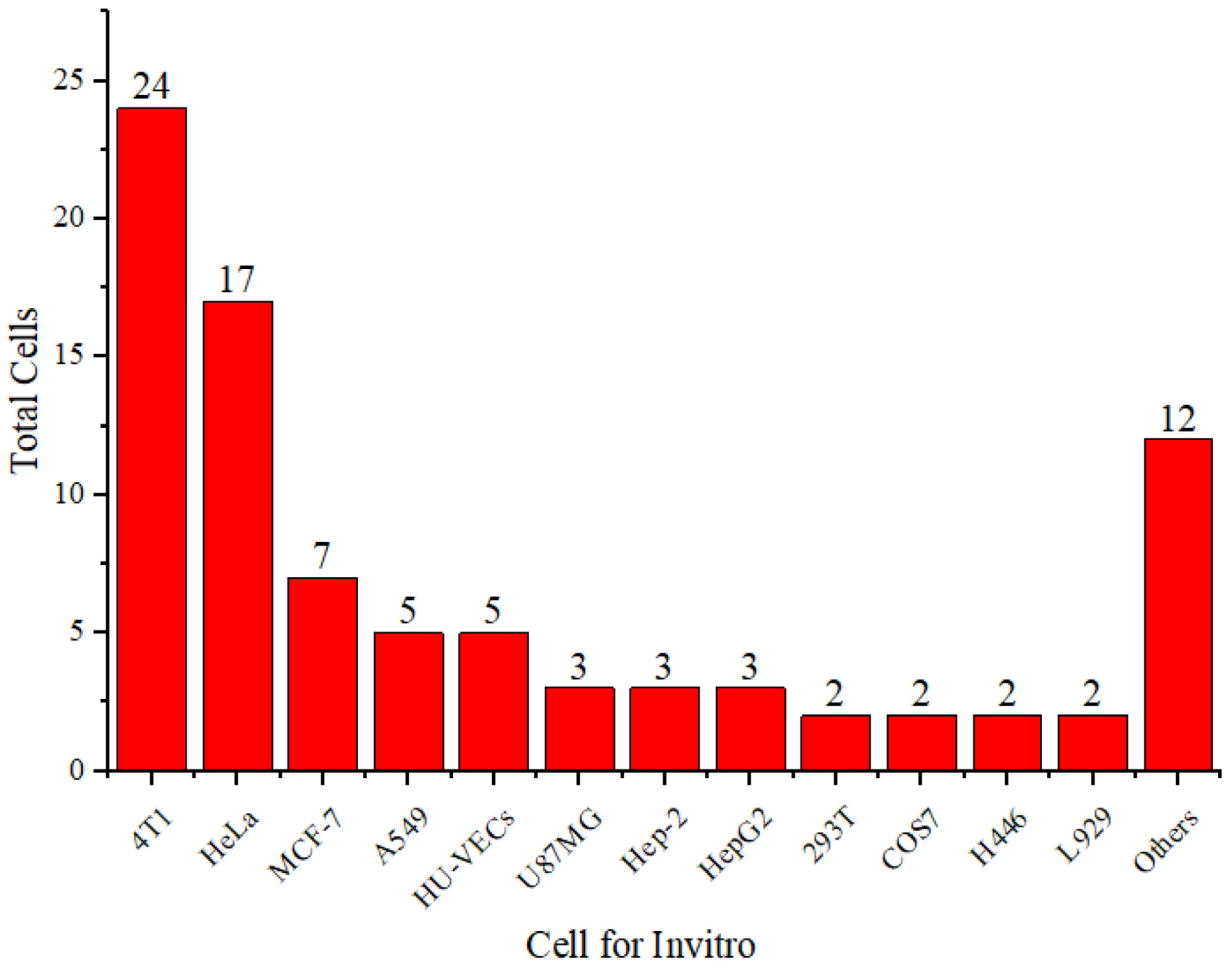
Distribution of cell types used *in vitro*.

**Figure 5** shows that 4T1 and HeLa cells are more frequently used than other cell types. The 4T1 cell line is a highly tumorigenic and invasive transplantable tumor cell line 70. Unlike many tumor models, 4T1 can spontaneously metastasize from the primary tumor in the mammary gland to several distant sites, including the lymph nodes, blood, liver, lungs, brain, and bones.

**Figure 6:**
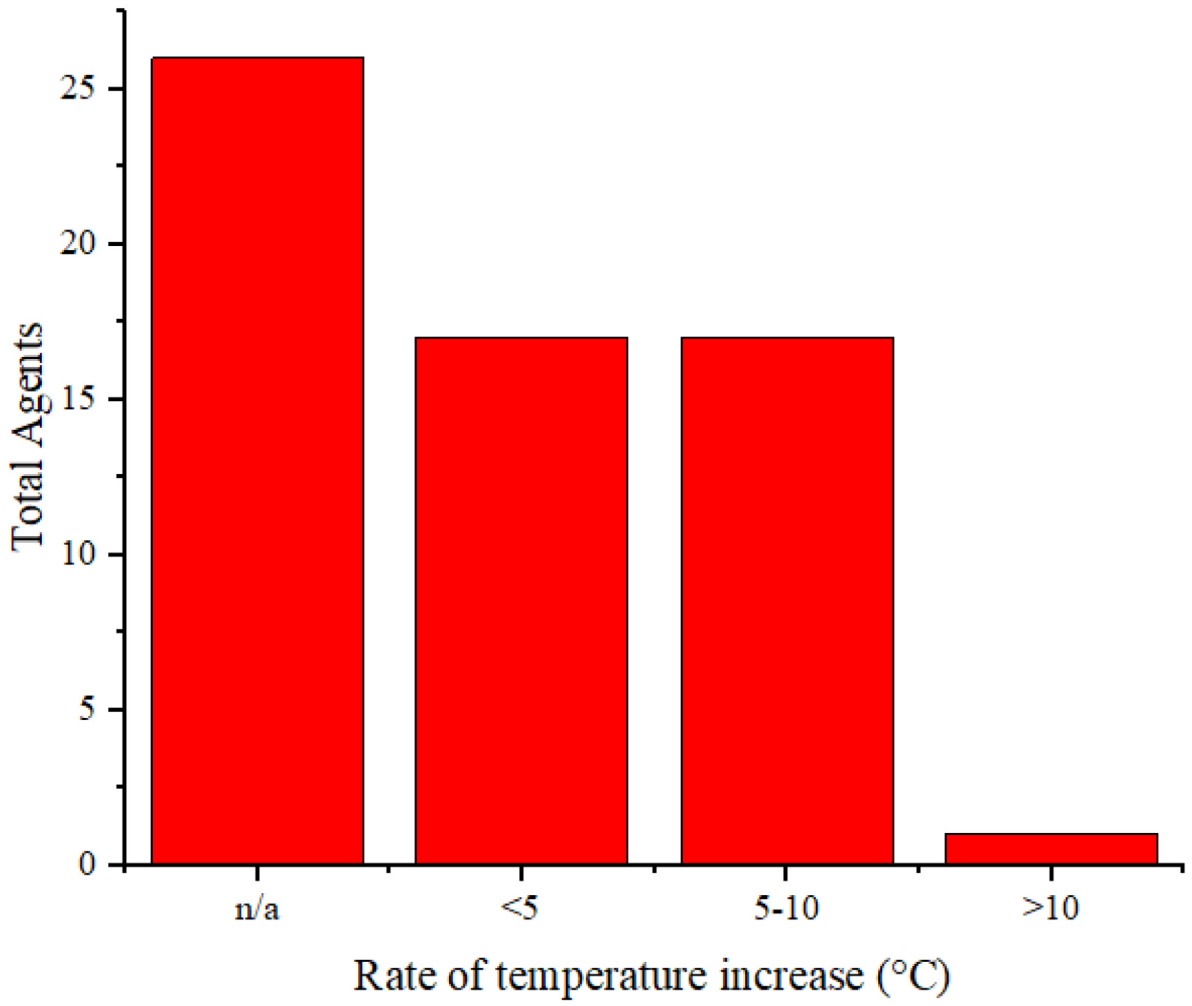
Distribution of temperature increase *in vitro* for the agents.

In addition to serving as contrast agents, these agents are used as cancer therapies through PTT. Therefore, these agents should reach temperatures sufficient to eliminate cancerous cells. Thermal cancer therapy is predominantly identified with temperatures ranging from 40 to 48°C sustained at the treated site for an hour or longer 71. Moreover, temperatures above 42°C lead to cell death in various tissues 72. Agents undergo laser irradiation for a specified duration to attain temperatures that can eradicate cancer cells. We cannot directly compare the effectiveness of the agents in temperature elevation because of varying radiation treatments (wavelengths, power, and duration). **Figure 6** illustrates the rate of temperature increase calculated from the temperature change data and radiation duration applied to the agents. The highest rate of temperature increase was achieved by rGO-AuNP at 12.2 °C/min using a radiation wavelength of 1061 nm (0.25 W/cm^2^, 300 s) 47. The exact reason why this agent exhibits a higher rate of temperature increase than other agents is not explicitly stated. Comparatively, for agents irradiated at the same radiation wavelength (NIR-II), Mo_2_C-derived polyoxometalate (POM) irradiated at 1060 nm (1 W/cm^2^, 300 s) achieves a rate of 7.4°C/min 53, and 2H-MoS_2_ irradiated at 1064 nm (1 W/cm^2^, 480 s) achieves a rate of 1.55 °C/min 21. Among agents irradiated in the NIR-I or visible light region, the highest rate of increase is observed in the PQs-TPA NPs agent irradiated at a wavelength of 808 nm (0.8 W/cm^2^, 300 s) at 9.2 °C/min 40. In the comparison of agent composition, rGO-AuNP containing Au, AuNSPHs, Au_44_MBA_26_-Cy_7_ NCs, Au@Cu_2_‑xSe NPs, J-Au-CS-QD, and Au-MUA-TMA exhibited significantly lower rates at 4°C/min, 6.7°C/min, 4°C/min, 2.83°C/min, and 2.8°C/min 18,39,45,46,60.

**Figure 7:**
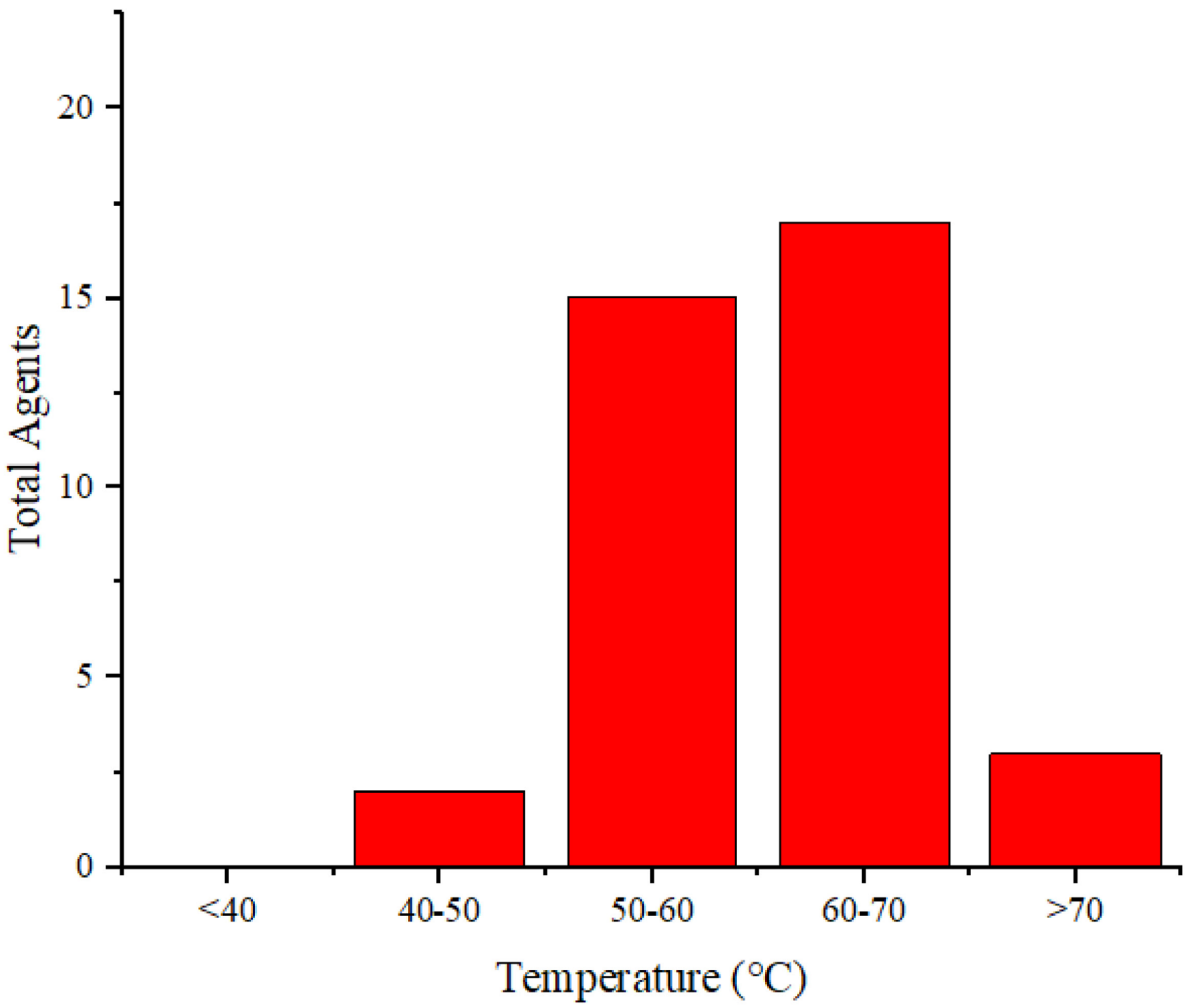
Distribution of the achieved temperature increase by the agents.

**Figure 7** illustrates the distribution of the maximum temperature increases for the agents. Referring to the 40°C-48°C temperature range for cancer therapy 71, nearly all these agents can be applied for cancer treatment. Silica@GNS achieved the highest temperature at 80°C when irradiated with a wavelength of 808 nm (1 W/cm^2^, 900 s) 63. Once again, we could not find a precise reason for this agent reaching a higher temperature than others. This necessitates a review of numerous factors such as pH, density, size, and even the composition of the agents.

**Figure 8:**
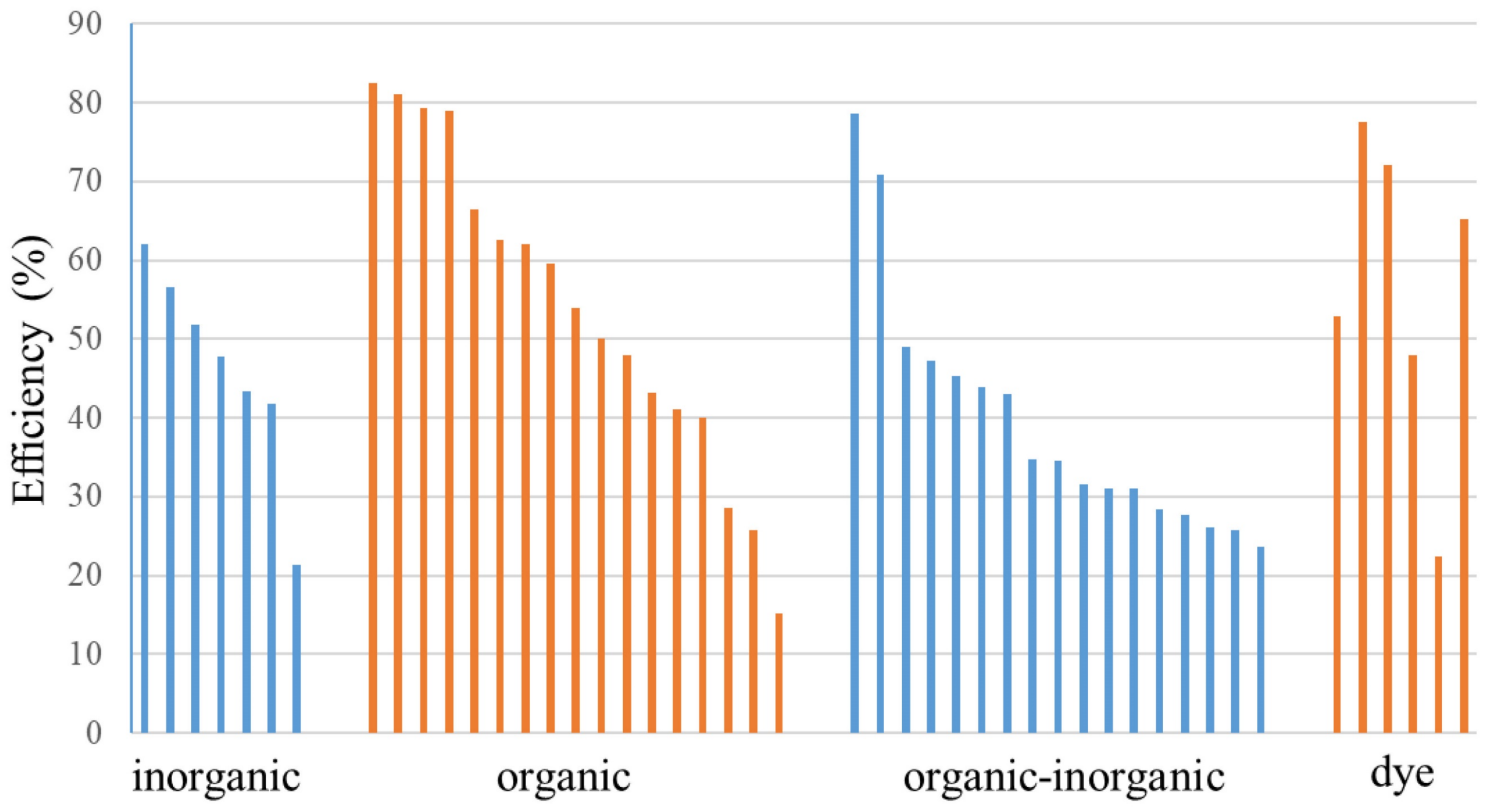
Photothermal conversion efficiency of agents based on their constituent components.

**Figure 9:**
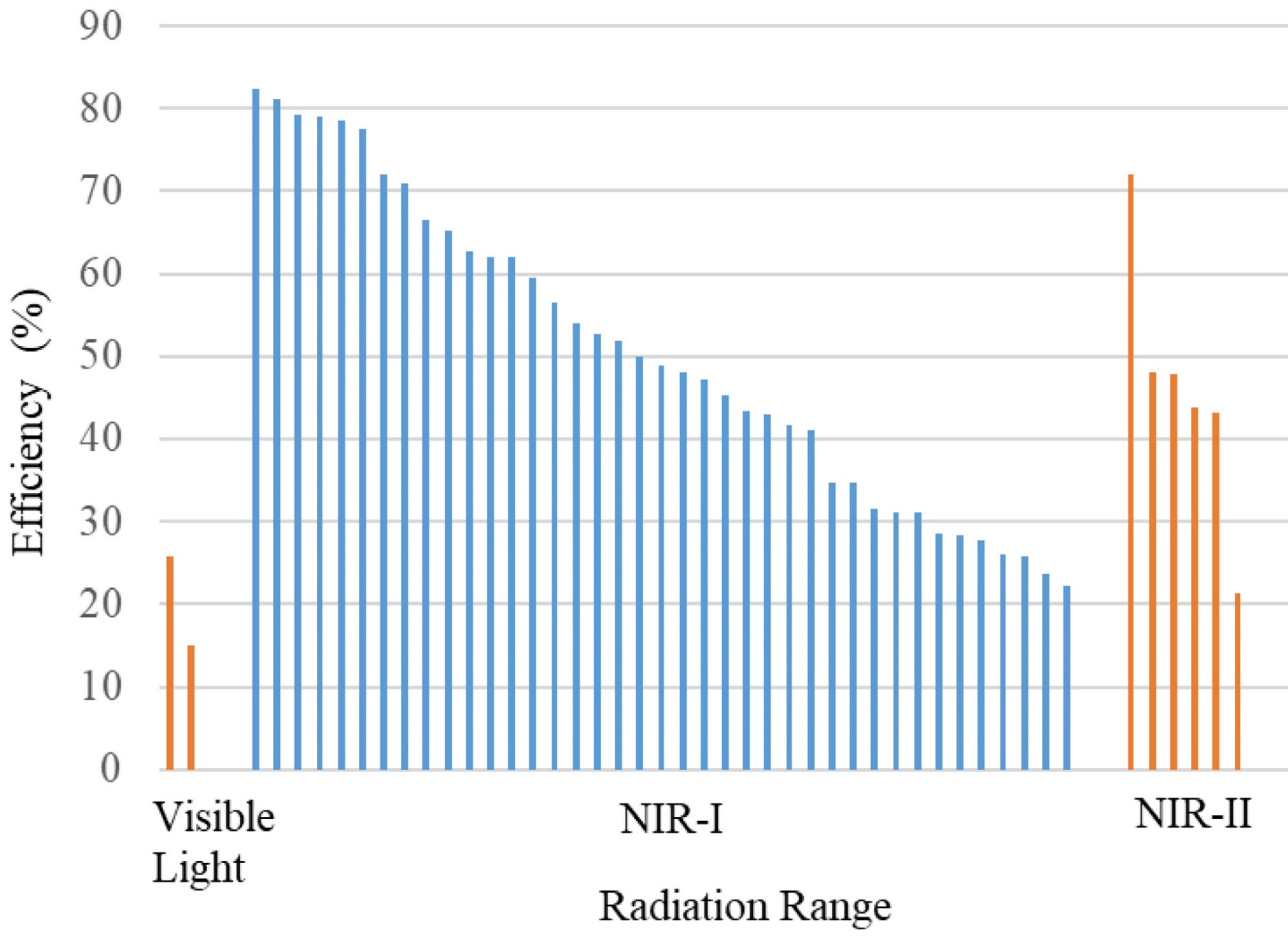
Photothermal conversion efficiency of agents based on their constituent components in radiation range.

**Figure 10:**
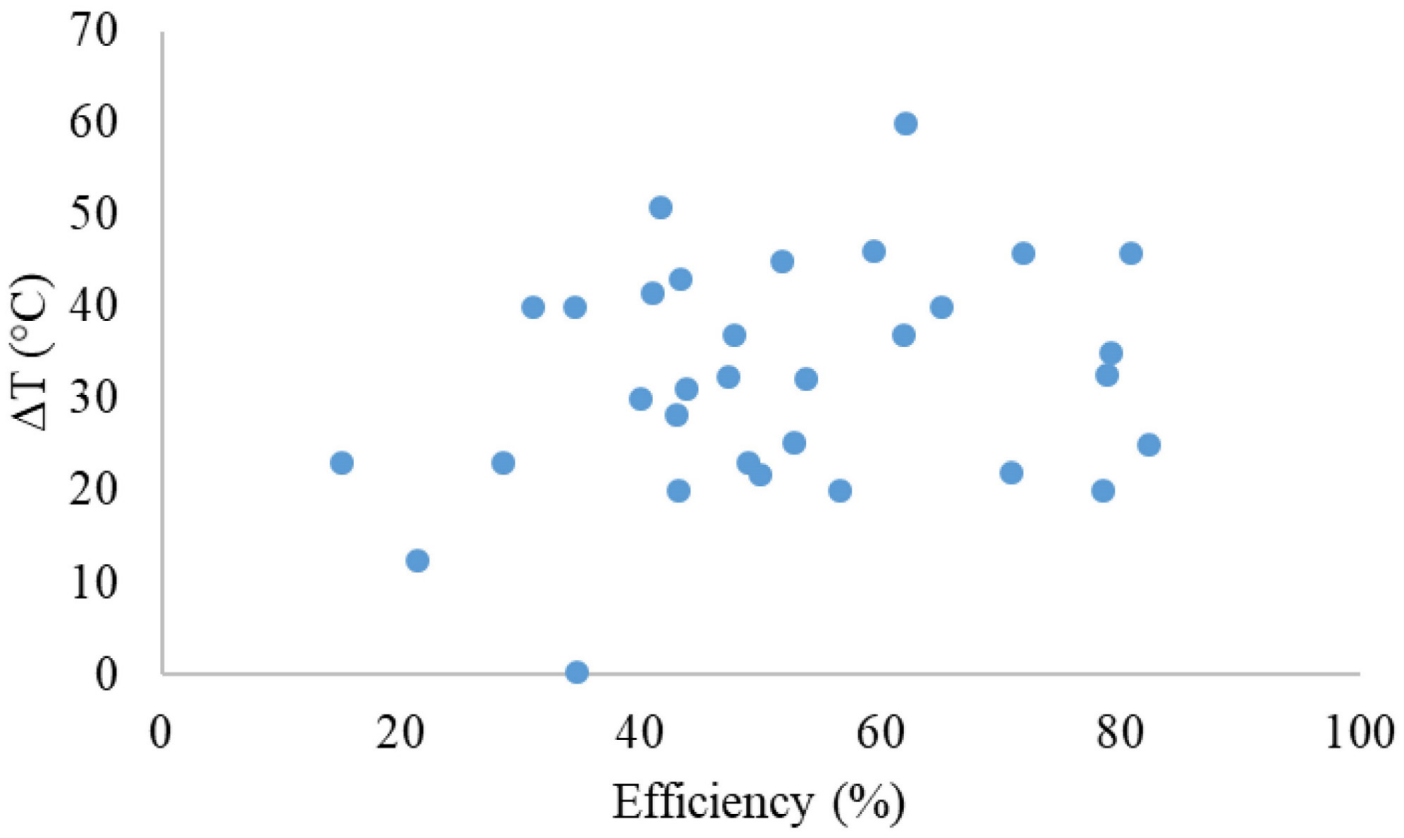
Relationship between photothermal conversion efficiency and temperature change of the agents.

**Figure 11:**
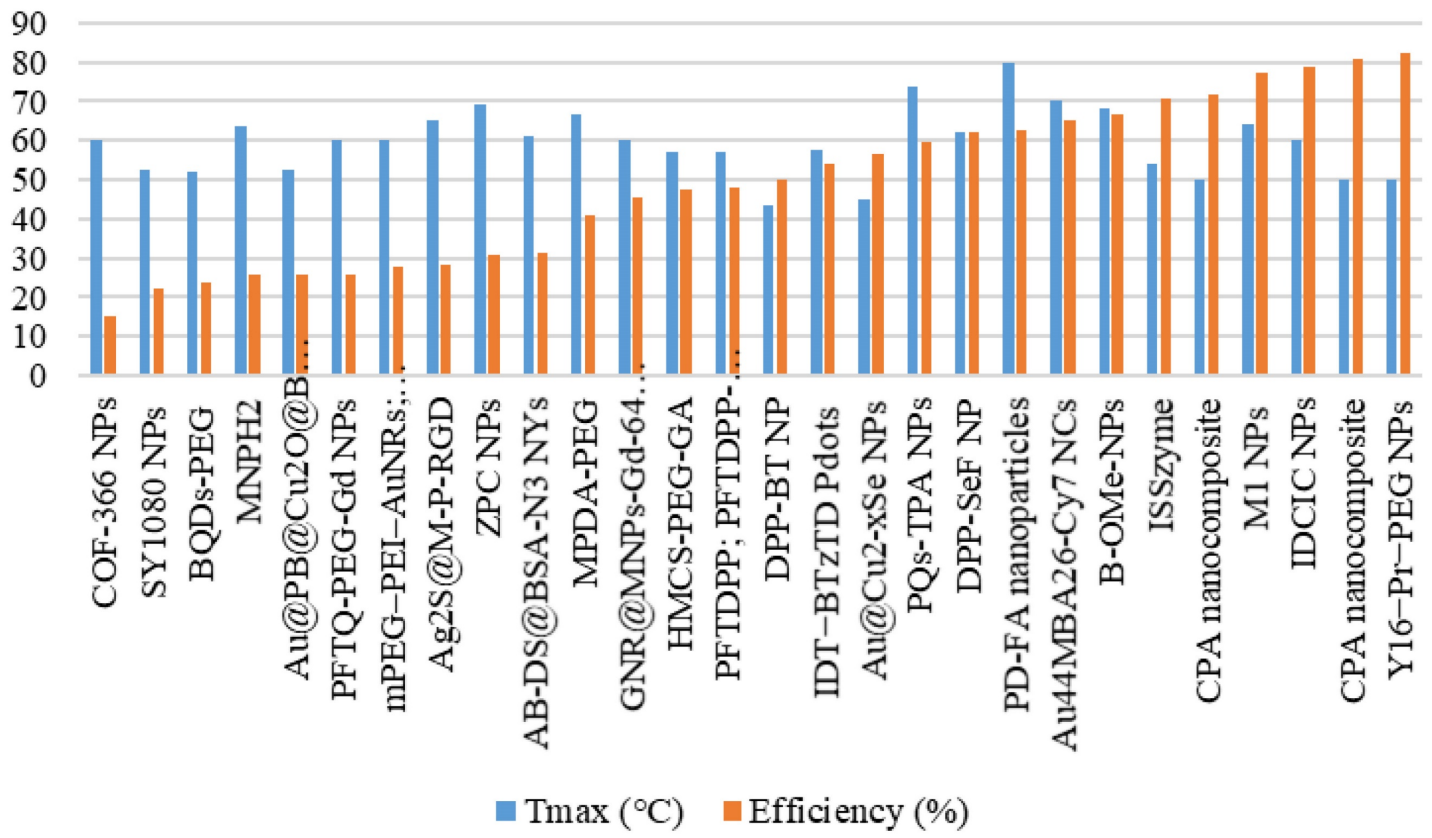
Agents' maximum attained temperature and photothermal conversion efficiency.

**Figure 8** shows the thermal conversion efficiency distribution based on the agent type. Photothermal conversion efficiency refers to the ability of an agent to convert light energy into heat. This graph compares conversion efficiency among various materials such as organic, inorganic, hybrid, or dyes. This data is essential for understanding how each component enhances the effectiveness of photothermal applications in cancer therapy or local heating. **Figure 9** shows the distribution of thermal conversion efficiency across different absorbance wavelength regions. This graph is used to determine the optimal efficiency at specific absorbance wavelength regions for medical applications. From these figures, we cannot conclude that the agent's components and the length of the radiation laser source directly affect the increase in photothermal conversion efficiency. Improving photothermal conversion facilitates enhanced efficiency in PTT and contributes to an increase in photoacoustic signals 18. **Figure 10** illustrates the relationship between photothermal conversion efficiency and the temperature change produced by the agent. The graph does not show a linear relationship between higher conversion efficiency and temperature increase. **Figure 11** depicts the relationship between photothermal conversion efficiency and the temperature the agent achieves. This graph provides insights into the potential of each agent to reach the high temperatures necessary for therapeutic applications.

This information helps evaluate and compare agents' performance under intensive use conditions. Various parameters, such as the laser intensity applied, can influence the efficiency and temperature increase. The intensity of the laser light affects the amount of energy absorbed by the photosensitizer or nanoparticles, which in turn influences the rise in temperature. Therefore, essential factors affecting photothermal performance and the methods and mechanisms of laser-induced photothermal energy must be studied 73.

Additionally, the volume fraction, shape, size, and material type of the agent compounds influence the efficiency and temperature rise achievable by the agent 74. The highest photothermal conversion efficiency is observed in Y16-Pr-PEG NPs, reaching 82.4%, irradiated at a wavelength of 808 nm (1 W/cm2, 300 s), resulting in a temperature increase of 25°C and achieving a temperature of 50°C 38. In comparison, MPDA-PEG, irradiated at a wavelength of 808 nm (2 W/cm2, 300 s) 55, exhibits a lower photothermal conversion efficiency of 41%, a temperature increase of 41.5°C, and a temperature of 66.5°C.

Although photothermal therapy is promising as an exact treatment method, challenges remain in improving photothermal conversion efficiency and achieving uniform heat distribution within the tumor. Uneven heat distribution can result in incomplete tumor ablation and potential damage to surrounding healthy tissues. Further research is needed to develop new materials with optimized optical and thermal characteristics to enhance the efficiency of photothermal therapy. These parameters include heating conditions (laser intensity, wavelength, radius, and heating time), nanoparticle parameters (volume fraction, shape, size, and material), and boundary conditions (cooling methods and cooling time), all of which are applied to control therapeutic outcomes (Ren et al., 2022).

### PTT using *in vivo* methods

**Table 4** summarizes the thermal characteristics of the agent compounds in the *in vivo* methods, including the implanted cells in experimental animals, thermal camera tools, laser irradiation, and temperature increase of the agents.

**Figure 12:**
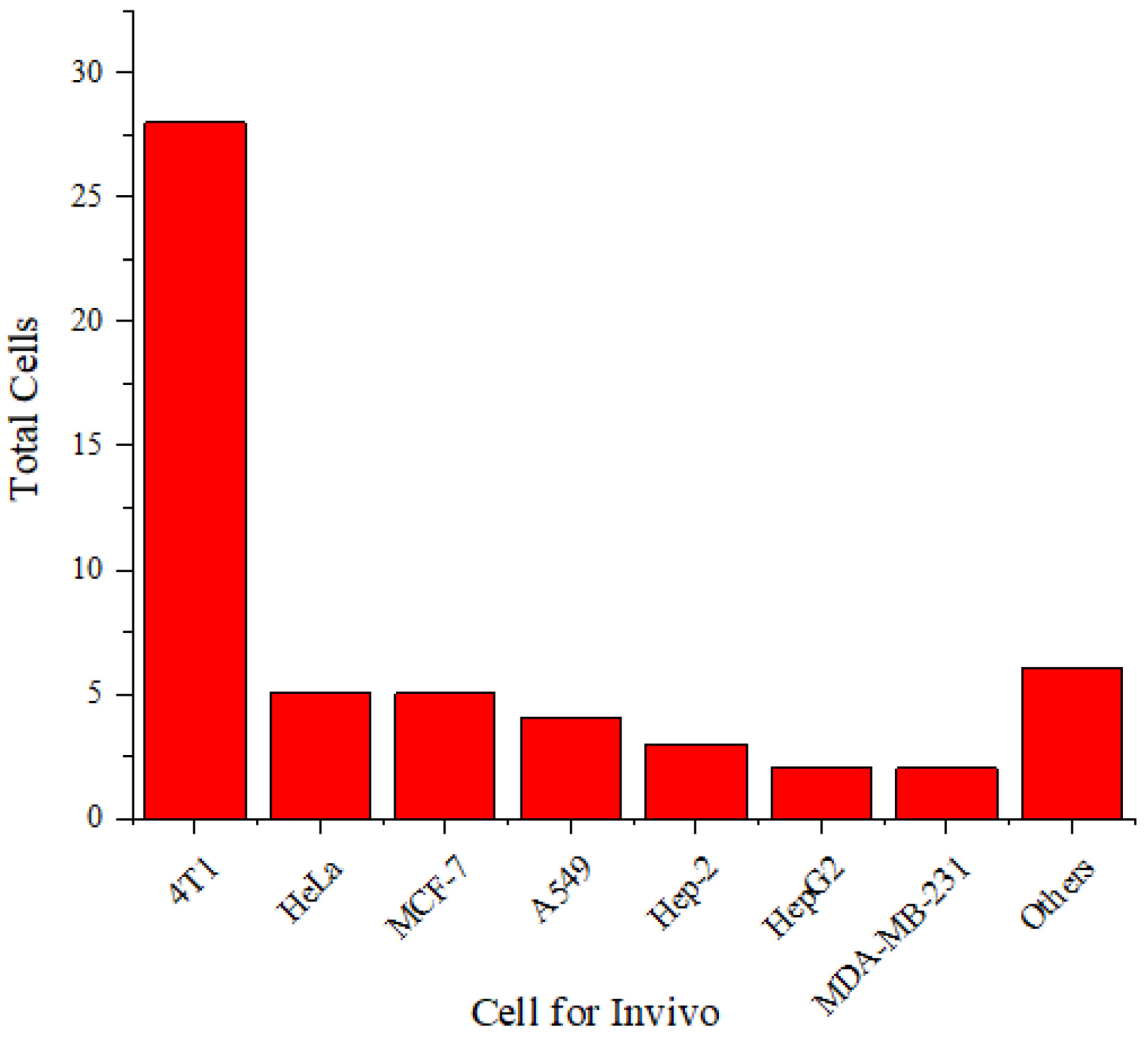
Distribution of cells used *in vivo*.

**Figure 12** shows that the 4T1 cell line is more frequently used in experimental animals than other cell lines. The 4T1 tumor exhibits several characteristics that make it a suitable animal model for human breast cancer 70. The progressive metastatic spread of 4T1 to lymph nodes and other organs is highly reminiscent of human breast cancer. The 4T1 tumor is also resistant to 6-thioguanine. This characteristic enables accurate counting of metastatic cells, even when spread at submicroscopic levels in distant organs.

**Figure 13:**
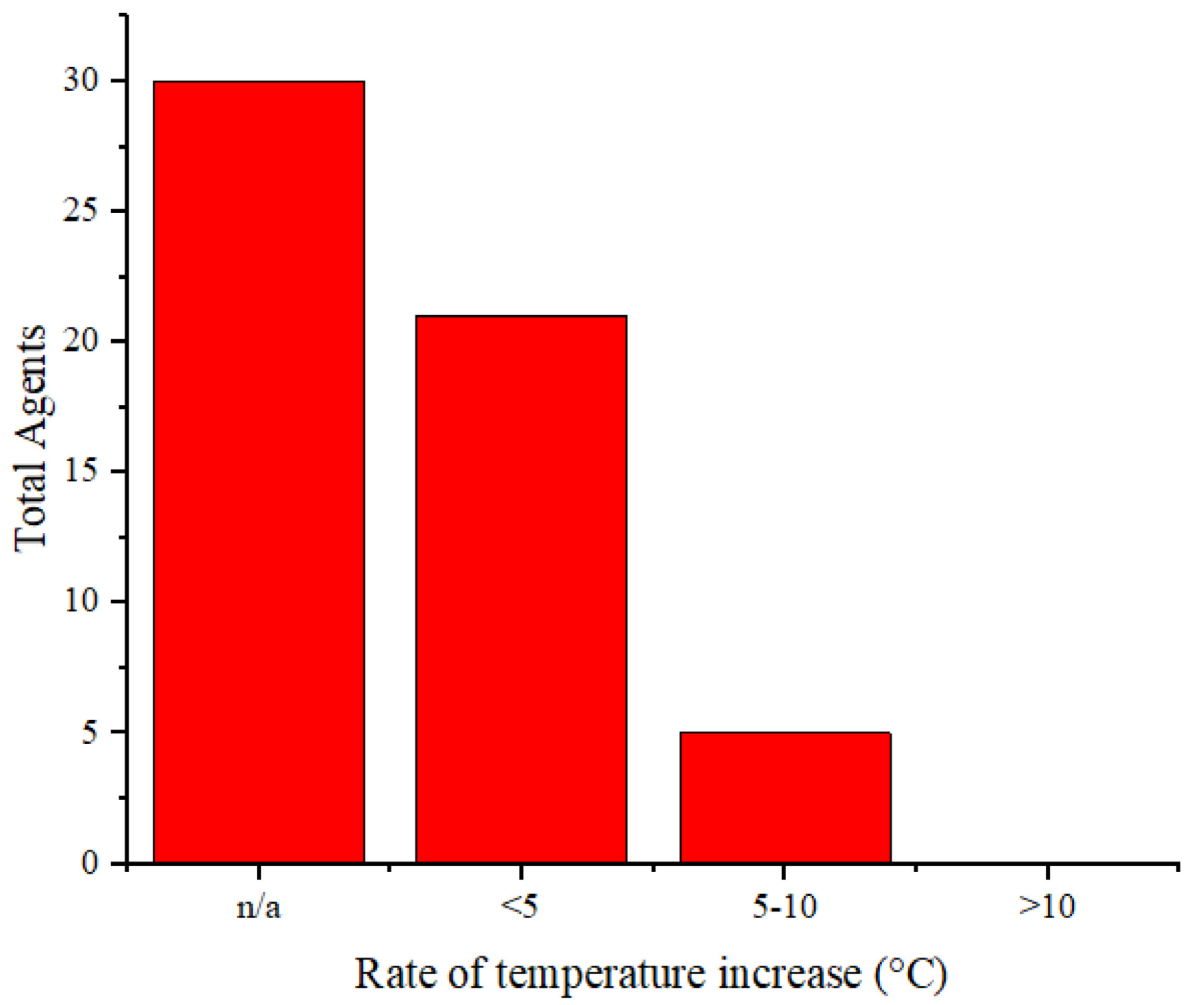
Distribution of the rate of temperature increase of the agents *in vivo*.

**Figure 14:**
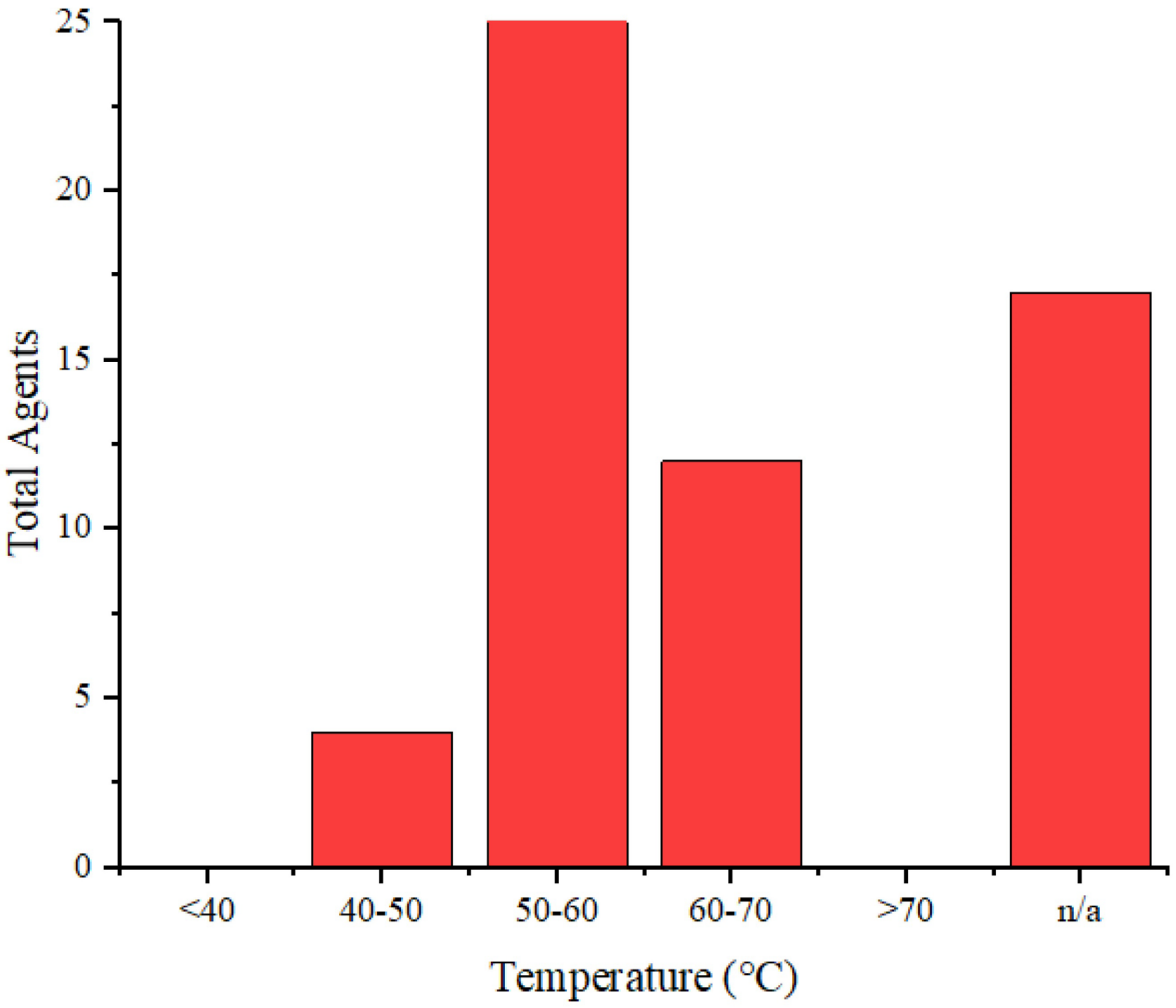
Distribution of temperatures achieved by the agents *in vivo*.

Similarly, as in *in vitro* studies, agents are used as a therapeutic agent for cancer implanted in experimental animals. These agents are injected into the experimental animals, accumulate in the cancer cells, and are then exposed to radiation, causing a sufficient increase in temperature to eliminate cancer cells. **Figure 13** illustrates the distribution of the rate of temperature increase of the agents *in vivo*, whereas **Figure 14** displays the distribution of the achieved temperature of the agents *in vivo*. Fe-ZDS at 7.5°C/min recorded the highest rate, with radiation at a wavelength of 808 nm (0.82 W/cm^2^, 360 s) 52. Meanwhile, 1T-MoS_2_ nanodots achieved the maximum temperature increase at 65.7°C using radiation at a wavelength of 808 nm (1 W/cm^2^, 480 s). **Figure 14** and **Table 4** also indicate agents that can achieve a temperature range of 40°C-48°C, which is helpful for cancer therapy 71.

**Figure 15:**
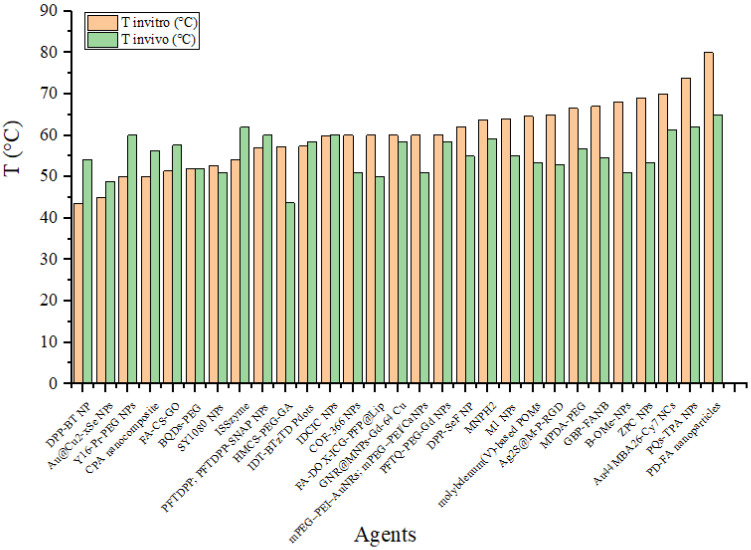
Comparison of temperatures achieved by the agents *in vitro* and *in vivo*.

**Figure 15** compares the agent's temperatures when irradiated *in vitro* and *in vivo*. This graph shows the differences in heating between the two conditions. This data is essential for understanding how the biological environment affects the agents' performance. It also helps assess whether laboratory results can be expected to be applied to practical applications in the human or animal body. A difference is observed in the agent's temperature when exposed to *in vivo* radiation, generally resulting in lower temperatures than when the agent is irradiated in an *in vitro* environment. This change may be due to the laser energy absorbed by the medium (experimental animal) before reaching the agent. However, some agents exhibit higher temperatures when tested *in vivo* than *in vitro*. For example, DPP-BT NP exhibited a temperature of 43.6°C at 100 μg/mL during *in vitro* testing and reached 54°C at 1 mg/mL in 200 µL *in vivo* 16. Furthermore, the ISSzyme agent reached a temperature of 54.17°C when irradiated at a wavelength of 808 nm (1 W/cm^2^, 600 s) *in vitro* and during *in vivo* irradiation at the same wavelength (808 nm) but with a power of 2 W/cm^2^ for 600 s. Under *in vivo* conditions, the temperature increase was due to treatment modifications, such as changes in the agent's volume and alterations in the power of the radiation source.

## Conclusion

Components used as therapeutic and contrast agents in photoacoustic imaging-guided PTT have been extensively developed, displaying variations in organic, inorganic, and hybrid organic-inorganic compounds. However, further development of dye-type agents is required. Generally, therapeutic and contrast agent development tends to exhibit absorption in the NIR-I region compared with that in the NIR-II region. Developing agents with NIR-II absorption can offer advantages, including improvement in the contrast of photoacoustic imaging. These agents were then tested for PTT *in vitro* and *in vivo*, with 4T1 cells being the most frequently used. When irradiated with specific power and duration, the agents experience a temperature increase of over 40°C, which is sufficient to eliminate cancer cells. No specific relationship was found between the photothermal conversion efficiency and the temperature increase achieved by the agents. Enhancing photothermal conversion not only facilitates improved efficiency in PTT but also elevates photoacoustic signals. Therefore, a deeper exploration of the influence of photothermal conversion efficiency from other perspectives is necessary. There is substantial potential for extensive research involving further exploration in developing agents that act as therapeutic agents and serve as contrast agents to enhance photoacoustic imaging. The development of agents in the NIR-II region holds significant potential because of limited research in this domain.

Future works need to be carried out to develop potential contrast agents that enhance image contrast in photoacoustic imaging and possess therapeutic solid properties. The development of multifunctional agents capable of simultaneously detecting, mapping, and treating diseases will be a primary focus of future research. Additionally, there is potential to improve photothermal conversion efficiency of contrast agents and achieve more uniform heat distribution. Future research should focus on developing new materials with optimized optical and thermal characteristics to enhance the efficiency of photothermal therapy. Although there has been a significant amount of promising preclinical research, broader clinical validation is still needed to ensure the safety and effectiveness of these agents in photoacoustic and photothermal therapy for human treatment.

Further clinical studies will provide a better understanding of the clinical potential of these techniques and accelerate their application in clinical practice. Additionally, challenges remain in translating photoacoustic imaging and photothermal therapy techniques from the laboratory to the clinical practice. Future research should focus on developing methods that can facilitate the integration of these techniques into everyday clinical practice, including the development of more affordable and user-friendly hardware and the creation of standardized clinical protocols. Integrating photoacoustic imaging with other imaging modalities (e.g., MRI, CT) and combining photothermal therapy with other treatment strategies (e.g., chemotherapy, immunotherapy) could provide more comprehensive diagnostic and therapeutic solutions. Therefore, future research is expected to significantly contribute to developing more effective and safe photoacoustic and photothermal therapies.

## Figures and Tables

**Table 1:** Summary of the optical characteristics of the agents and imaging modalities

Agent	Type	λ_abs_ (nm)	λ_PA_ (nm)	Imaging modality	PAI tools	Ref
Polydopamine-coated mesoporous silica-gold nanorods (PDA-AuNRs@MSN)	Organic-inorganic	450	875	PAI	Vevo LAZR	15
Diketopyrrolopyrrole benzothiadiazole nanoparticles (DPP-BT NP)	Organic Semiconducting polymer	686	808; 730	Fluorescent; PA	Nexus 128	16
Polyethylene glycol- and polyethyleneimine-modified gold nanorods (mPEG-PEI-AuNRs); mPEG-PEI and calcium carbonate nanoparticles (mPEG-PEI/CaNPs)	Organic-inorganic	n/a	n/a	Fluorescent; PAI	MSOT InVision 128	17
Gold, 11-mercaptoundecanoic acid, and 10-mercaptodecyl) trimethylammonium bromide nanoparticles (Au-MUA-TMA)	Organic-inorganic	524	n/a	PAI	MSOT InVision 128	18
Benzobisthiadiazole (BBTD-1302)	Organic semiconducting polymer	942	1302; 942	Fluorescent; PAI	MSOT imaging system	19
Emeraldine salt of polyaniline in sodium bis(2-ethylhexyl) sulfosuccinate (PANI-ES@AOT)	Organic-inorganic	n/a	808; 970	PAI	Vero LAZR-X mX550D	20
1T Molybdenum disulfide (1T-MoS_2_); 2H Molybdenum disulfide (2H-MoS_2_)	Inorganic	1300; 427, 606, 653	1280	PAI	n/a	21
Benzodithiophene-Thieno[3,4-b]thiophene (BDT-TH); Benzodithiophene-Naphthalenediimide (BDT-NDI)	Organic Semiconducting polymer	516; 700	532; 700	PAT	Homemade PA system	22
Boron-dipyrromethene-Fe nanoparticles (BDP-Fe NPs)	Organic-inorganic	1300	1064	PAI	n/a	23
3-azidopropylamine (-N_3_^-^) with Bi_2_S_3_-Ag_2_S-DATS@BSA nanoparticles (AB-DS@BSA-N_3_ NYs)	Organic-inorganic	900	808	Fluorescent; PAI	MSOT; II900/1700 *in vivo* imaging System (808 nm, 150 ms)	24
Hollow mesoporous carbon spheres-polyethylene glycol-gambogic acid (HMCS-PEG-GA)	Organic-inorganic	808	715	PAI	High-resolution preclinical photoacoustic system	25
Melanin nanoparticles H_2_ (MNPH_2_)	Organic	457	808	PAI	The MSOT scanner consists of a range of 680-980 nm	26
Folic acid-conjugated chitosan-functionalized graphene oxide (FA-CS-GO)	Organic-inorganic	578, 720	532	PAI	PAM system (using a Q-switched diode-pumped solid-state laser (SPOT-10-100-532) and a commercially focused transducer (V324-SM); a reflection-mode fluorescent imaging system	27
Diketopyrrolopyrrole (DPP) was copolymerized with strong electron-donating substitutes (fluorene and thiophene) (PFTDPP); PFTDPP combined with S-nitroso-N-acetylpenicillamine (PFTDPP-SNAP NPs)	Organic	350-500, 550-900	808	Fluorescent; PAI	PA imaging	28
Porphyrin-based covalent organic framework nanoparticles (COF-366 NPs)	Organic	590, 297	n/a	PAI	MSOT InVision 128	29
CoCuFe-selenide (CCFS)-polyvinyl pyrrolidone (PVP)-L-arginine (L-Arg) (CPA nanocomposite)	Organic	808	n/a	PAI	MSOT InVision 128	30
Squaraine dye nanoparticles (SQ-NPs)	Dye	600-800	760	PAI	n/a	31
Molybdenum(V)-based polyoxometalate (POMs)	Inorganic	1065	1064	PAI	MSOT InVision 128	32
Cyclo(Arg-Gly-Asp-Dphe-Lys) @ polymer dithienopyrrole -thiadiazolobenzotriazole (cRGD@PT NPs)	Organic	1000	1064	PAI	LOS-3D Photoacoustic imaging	33
ISSzyme	Organic-inorganic	700	532	PAI	Homemade PACT system (Q-switched Nd: YAG laser (532 nm))	34
SY1080 NPs	Organic dye	820	808	Fluorescent; PAI	MSOT imaging system	35
Bodipy-^1^O_2_ methoxyl (B-OMe-NPs)	Organic	735	809	Fluorescent; PAI	n/a	36
PEG-modified boron quantum dots (BQDs-PEG)	Organic-inorganic	n/a	808	Fluorescent; PAI	n/a	37
Y16-Pr-PEG NPs	Organic	800	808	Fluorescent; PAI	Confocal laser scanning microscopy (Leica SP8); Near-infrared InGaAs array imaging detector (EX, 808 nm; 2 W/cm2)	38
Gold nanostar polyaniline hyaluronic acid (AuNSPHs)	Organic-inorganic	n/a	850	PAI	VisualSonics Vevo LAZR	39
pyrazino[2,3g]quinoxaline (PQs)-TPA NPs	Organic small molecules	675	760	Fluorescent; PAI	MSOT InVision 128	40
Melanin-nanoparticular polydopamine-indocyanine green (Mel-pDA-ICG)	Organic dye	808	808	Fluorescent; PAI	VEVO LAZR-X (at a wavelength of 680-970 nm	41
Pt/Te nanoheterostructures (PT); mPT	Inorganic	n/a	744	PAI	744 nm, 120 nJ laser by the high-resolution PAI system)	42
Hemicyanine dye nanoparticles (M1 NPs)	Organic dye	734, 1040	734; 1040	Fluorescent; PAI	Perkin Elmer IVIS Lumina III; PACT scanner (inVision256-TF)	43
PEGylated Sb-835 NPHs	Inorganic	835	835	Fluorescent; PAI	n/a	44
Au-MBA denotes 4-mercaptobenzoic acid-Cy7 (Au_44_MBA_26_-Cy7) NCs	Organic-inorganic dye	497, 590, 794	808	Fluorescent; PAI	n/a	45
Janus chitosan/gold quantum dots (J-Au-CS-QD)	Organic-inorganic	808	690	Fluorescent; PAI	n/a	46
Gold nanoparticle-coated reduced graphene oxide (rGO-AuNP)	Organic-inorganic	1000	1250	US + PAI	VisualSonic Vevo 2100 LAZR (40 MHz)	47
MoSe_2-x_ nanoflowers (MNFs)	Inorganic	n/a	800	PAI	Endra Nexus 128	48
W_18_O_49_ nanorods	Inorganic	n/a	n/a	PAI	MSOT imaging system	49
Polymer PIIGDTS nanoparticles with folic acid modification (PD-FA)	Organic	450, 845	845	PAI	MSPAM system (wavelength tunable nanosecond pulsed OPO laser (Continuum, USA), ultrasound transducer with a central frequency of 25 MHz (Olympus, USA))	50
Diketopyrrolopyrrole (DPP)-SO; DPP-SS; DPP-SSe NPs	Organic	900; 912; 960		PAI	MSOT imaging system	51
Fe^3+^ complex Zwitterionic dopamine sulfonate (Fe-ZDS)	Organic-inorganic	450-900	680, 808	MRI; PAI	PA imager under the excitation of 680 or 808-nm pulse lasers	52
Mo_2_C-derived polyoxometalate (POM)	Inorganic	1060	n/a	PAI	NIR-II PAI images	53
DPP-SS; DPP-OF; DPP-SF; DPP-SeF NP	Organic	726; 735; 816; 820	n/a	PAI	MSOT InVision 128	54
PEG-modified polydopamine (MPDA-PEG)	Organic	n/a	n/a	PAI	High-resolution preclinical PAI system	55
TMB-F4TCNQ; TMB-TCNQ	Organic dye	400; 392	1300	PAI	PA images under 1300-nm laser excitation	56
IDCIC NPs	Organic	682	780; -1000	Fluorescent; PAI	FLI (FC-W-980-30W); PACT Scanner (Invision256-TF)	57
Glypican-3-binding peptide-Fe_3_O_4_ Core/Au shell nanocomplex (GBP-FANB)	Inorganic	520-680	640	Fluorescent; PAI	FLI (IVIS Lumina II (Excitation Filter: 640 nm, Emission Filter: 700 nm); PA (Endra Nexus128)	58
Indacenodithiophene (IDT)-BTzTD Pdots	Organic	800	n/a	CT, fluorescent; PAI	SuperArgus (with the following parameters: 50 kV, 300 μA, 33-μm resolution, and 300 ms of exposure time) PAI: homemade PAI system	59
Au@Cu_2_‑xSe NPs	Inorganic	NIR	808	CT; PAI	VEVO LAZR-X	60
FA-doxorubicin (DOX)-indocyanine green (ICG)-perfluoropentane (PFP)@Lip	Organic	450	n/a	US; PAI	n/a	61
Manganese pentacarbonyl bromide-loaded g-carbon nitride/polypyrrole (MnCO@CNPpy)	Organic-inorganic	410-430	n/a	PAI	Vevo LAZR-X (40 MHz)	62
Gold nanostars (GNS); Silica@GNS	Organic-inorganic	850	850	PAI	PA system (OPO laser 850 nm; hydrophone (HNC-1500, ONDA, Sunnyvale, CA); amplifier (5072PR, OLYMPUS, Waltham, MA); transducer (Immersion Transducers, Olympus Corp., Tokyo, Japan) with a center frequency of 5 MHz; TWPA-I	63
ZrO_2-x_@PEG/Ce6 (ZPC NPs)	Organic-inorganic	~405; ~660	n/a	Fluorescent; PAI	Fusion FX Spectra; Vevo LAZR-X multimodal imaging system	64
Gold nanorod melanin nanoparticles (GNR@MNPs)-Gd-64 Cu	Organic-inorganic	700-900	680-980	PET; MRI; PAI	Siemens Inveon micro PET; 3.0-T MR scanner; MSOT inSight 128 (under a 680-980 nm pulse laser);	65
PFTQ-PEG-Gd NPs	Organic-inorganic	760	760	FLI; MRI; PAI	CDD: NIRvana TE 640; micro-MRI scanner (0.5 T, NIUMAG, NMI20-015 V-I, TE/TR = 18/120 ms); Vevo LAZR PAI System	66
Ag_2_S@M-P-RGD	Organic-inorganic	275	744	FLI; PAI	n/a	67
Au@PB@Cu_2_O@BPQDs/PAH NCs	Organic-inorganic	650-950	514; 650	MRI; PAI	3.0 T clinical MRI system; PAI n/a	68

PS: n/s signifies that the information provided is not written or not detailed.

**Table 2:** Summary of agent distribution based on their absorbance wavelength regions

λ_abs_ (nm)	Agents
Visible region (400-700 nm)	PDA-AuNRs@MSN 15; DPP-BT NP 16; Au-MUA-TMA 18; 2H-MoS_2_ 21; BDT-TH; BDT-NDI 22; MNPH_2_ 26; FA-CS-GO 27; PFTDPP 28; COF-366 NPs 29; ISSzyme 34; PQs-TPA NPs 40; Au_44_MBA_26_-Cy7 NCs 45; PD-FA 50; Fe-ZDS 52; TMB-F4TCNQ; TMB-TCNQ 56; IDCIC NPs 57; GBP-FANB 58; DOX- ICG-PFP@Lip 61; MnCO@CNPpy 62; ZPC NPs 64; Ag_2_S@M-P-RGD 67;
NIR-I (700-1000 nm)	BBTD-1302 19; AB-DS@BSA-N_3_ NYs 24; HMCS-PEG-GA 25; FA-CS-GO 27; PFTDPP-SNAP NPs 28; CPA nanocomposite 30; SQ-NPs 31; SY1080 NPs 35; B-OMe-NPs 36; Y16-Pr-PEG NPs 38; Mel-pDA-ICG 41; M1 NPs 43; PEGylated Sb-835 NPHs 44; Au_44_MBA_26_-Cy7 NCs 45; J-Au-CS-QD 46; PD-FA 50; DPP-SO; DPP-SS; DPP-SSe NPs 51; Fe-ZDS 52; DPP-SS; DPP-OF; DPP-SF; DPP-SeF NP 54; IDT-BTzTD Pdots 59; Au@Cu_2_‑xSe NPs 60; GNS; Silica@GNS 63; GNR@MNPs-Gd-64 Cu 65; PFTQ-PEG-Gd NPs 66; Au@PB@Cu_2_O@BPQDs/PAH NCs 68
NIR-II (1000-1700 nm)	1T-MoS_2_ 21; BDP-Fe NPs 23; POMs 32; cRGD@PT NPs 33; M1 NPs 43; rGO-AuNP 47; POM 53;

**Table 3:** Summary of the effects of photothermal agents *in vitro*

Agents	Cells	Therapy	λ_Rad_ (nm)	Power Rad (W/cm^2^)	Time Rad (s)	ΔT (°C)	T_max_ (°C)	Efficiency (%)	Ref
PDA-AuNRs@MSN	4T1	PTT	808	1	600	31.4	n/a	34.71	15
DPP-BT NP	HeLa	PDT; PTT	730	1	600	21.8	43.6	50	16
mPEG-PEI-AuNRs; mPEG-PEI/CaNPs	MCF-7, HeLa, 293T	UST; PTT	808	1.5; 0.8	600; 360	n/a	60	27.7	17
Au-MUA-TMA	U87MG	PTT	808	1	600	28	65	n/a	18
BBTD-1302	4T1	PTT	980	1	n/a	23	n/a	28.6	19
PANI-ES@AOT	HeLa, MCF-7	PTT	1064	1	300	31.1	n/a	43.9	20
1T-MoS_2_ nanodots; 2H-MoS_2_	A549; HeLa	PTT	808; 1064	1	480	43; 12.4	n/a	43.3; 21.3	21
BDT-TH; BDT-NDI	MCF-7	(LED) PTT	660	0.2	600	25-30	n/a	40	22
BDP-Fe NPs	HeLa	PTT	730	n/a	600	23	n/a	49	23
AB-DS@BSA-N_3_ NYs	Hep-2	PTT	808	1	300	n/a	60.9	31.6	24
HMCS-PEG-GA	HepG2	PTT	808	1	300	32.3	57.3	47.3	25
MNPH2	Hep-2	PTT	808	0.75	300	n/a	63.8	25.7	26
FA-CS-GO	MDA-MB-231	PTT	808	2	300	n/a	51.4	n/a	27
PFTDPP; PFTDPP-SNAP NPs	MCF-7	PTT	808	1	300	n/a	57	48	28
COF-366 NPs	4T1	PDT; PTT	635	0.2; 1.5	300	23	60	15.07	29
CPA nanocomposite	U87mg, HeLa, HepG2, COS7	PTT	808	1	600	12.9-45.9	50.1	72 (pH 7.4) 81 (pH 5.4)	30
SQ-NPs	MCF-7; A549	PTT	760	0.5	600	25.1	n/a	52.8	31
Molybdenum(V)-based POMs	4T1	PTT	1064	0.8	300	n/a	64.6	n/a	32
cRGD@PT NPs	U87MG, C6, GL261, HU-VECs, and HeLa	PTT	1064	1	300	20	n/a	43.15	33
ISSzyme	4T1; HU-VECs	PTT	808	1	300	32.2	54.17	70.9	34
SY1080 NPs	4T1	PTT	808	1	300	n/a	52.7	22.3	35
B-OMe-NPs	4T1	PTT	735	1	600	n/a	68	66.5	36
BQDs-PEG	4T1	PTT	808	1	600	n/a	52	23.7	37
Y16-Pr-PEG NPs	4T1	PTT	808	1	300	25	50	82.4	38
AuNSPHs	4T1; bEnd.3	PTT	809	1	300	20	n/a	78.6	39
PQs-TPA NPs	L929, HeLa, 4T1	PTT	808	0.8	300	46	73.8	59.5	40
Mel-pDA-ICG	4T1, CT26	PTT	808	1.2	180	28	n/a	n/a	41
PT; mPT	HU-VECs, 4T1	PTT	808	1.5	600	45	n/a	51.84	42
M1 NPs	4T1	PTT	808	1	600	n/a	64	77.5	43
PEGylated Sb-835 NPHs	4T1,	PTT	808	1	540	60	n/a	62.1	44
Au_44_MBA_26_-Cy7 NCs	HeLa,	PTT	808	1.5	360	40	70	65.12	45
J-Au-CS-QD	4T1, HEK293	PTT	808	0.5	600	28.3	n/a	43	46
rGO-AuNP	SKOV-3	PTT	1061	0.25	300	61	n/a	n/a	47
MNFs	L929; 4T1	PTT	808	1	600	50.9	n/a	41.7	48
W_18_O_49_ nanorods	4T1; HU-VECs	CDT; TT	n/a	n/a	n/a	16.6	n/a	n/a	49
PD-FA nanoparticles	HeLa; cos7	PTT	845	1	600	n/a	80	62.6	50
DPP-SO; DPP-SS; DPP-SSe NPs	A549; H446	PTT	808	0.8	300	35	n/a	79.3	51
Fe-ZDS	EMT-6; MCF-7	PTT	808	1.65	300	40	n/a	30.99	52
POM	HUVEC, HeLa	CDT; PTT	1060	1	300	37	n/a	47.8	53
DPP-SS; DPP-OF; DPP-SF; DPP-SeF NP	A549; H446	PTT	808	0.75	300	-; -; -; 37	50, 52, 56, 62	62	54
MPDA-PEG	HEK-293T; A549	PTT	808	2	300	41.5	66.5	41	55
TMB-F4TCNQ; TMB-TCNQ	4T1, HeLa	PTT	1060	1	300	n/a	n/a	`; 48	56
IDCIC NPs	4T1	PTT	808	1	300	32.6	59.9	78.9	57
GBP-FANB	HepG2; PC3	PTT	630	1	600	n/a	67	n/a	58
IDT-BTzTD Pdots	HeLa	PTT	808	0.8	600	32.1	57.4	53.9	59
Au@Cu_2_‑xSe NPs	4T1	PTT	808	1	300	20	45	56.6	60
FA-DOX-ICG-PFP@Lip	Y79; Hu-VECs	PTT	808	1	300	>40	>60	n/a	61
MnCO@CNPpy	HeLa, MCF-7, L02	PTT	808	1	300	40	n/a	34.6	62
GNS; Silica@GNS	MSC	PTT	808	1	900	55	80	n/a	63
ZPC NPs	HeLa; 4T1	PDT; PTT	660; 808	0.76; 2	300	n/a	69.1	30.99	64
GNR@MNPs-Gd-64 Cu	Hep-2	PTT	808	1	300	n/a	60	45.3	65
PFTQ-PEG-Gd NPs	4T1	PTT	808	1	600; 300	n/a	>60	26	66
Ag_2_S@M-P-RGD	HeLa	PTT	808	2	600	n/a	65	28.35	67
Au@PB@Cu_2_O@BPQDs/PAH NCs	HeLa	PDT; PTT	650	1.5	300	n/a	52.5	25.73	68

PS: n/a signifies that the information provided is not written or not detailed.

**Table 4:** Summary of the photothermal effects of the agents *in vivo*

Agents	Cells	Therapy	Tools	λ_Rad_ (nm)	Power (W/cm^2^)	Time Rad (s)	ΔT (°C)	T_max_ (°C)	Ref
PDA-AuNRs@MSN	4T1	PTT	n/a	808	1	600	n/a	61.6	15
DPP-BT NP	HeLa	PDT; PTT	FLIR E50;	730	1	600	n/a	54	16
mPEG-PEI-AuNRs; mPEG-PEI/CaNPs	MCF-7	UST; PTT	838A-H-O-S; FLIR E5	808	0.8	360; 600	n/a	51	17
Au-MUA-TMA	U87MG	PTT	n/a	808	1	600	7	n/a	18
BBTD-1302	4T1	PTT	n/a	980	1	n/a	19	51	19
PANI-ES@AOT	MCF-7	PTT	IR camera	808; 1064	1	300	9.47	46.14	20
1T-MoS_2_ nanodots; 2H-MoS_2_	A549	PTT	IR thermal imaging camera	808; 1064	1	480	28.3; 12	65.7; -	21
BDT-TH; BDT-NDI	MCF-7	(LED) PTT	IR thermal camera	660	0.2	600	n/a	53	22
BDP-Fe NPs	HeLa	PTT	n/a	730	n/a	600	16.9	54	23
AB-DS@BSA-N_3_ NYs	Hep-2	PTT	Fluke Ti400	808	1	300	17.5		24
HMCS-PEG-GA	HepG2	PTT	IR imaging camera	808	1	300	14.7	43.7	25
MNPH2	Hep-2	PTT	IR thermal camera	808	1	300	25	59.2	26
FA-CS-GO	MDA-MB-231	PTT	IR thermal camera	808	2	300	n/a	57.6	27
PFTDPP; PFTDPP-SNAP NPs	MCF-7	PTT	n/a	808	1	600	28	60	28
COF-366 NPs	4T1	PDT; PTT	IR thermal camera	635	1.5	300	n/a	51	29
CPA nanocomposite	4T1	PTT	Fluke Ti480	808	1	600	17.6	56.2	30
SQ-NPs	4T1	PTT	n/a	760	0.6	900	12		31
molybdenum(V)-based POMs	4T1	PTT	Fluke Ti400	1064	1.8	300	23	53.5	32
cRGD@PT NPs	C6; GL261	PTT	IR thermal camera	1064	1; 0.5	300	n/a	54	33
ISSzyme	4T1	PTT	IR radiation Thermal camera	808	2	600	n/a	62	34
SY1080 NPs	4T1	PTT	Fluke Ti400	808	1	300	19	51	35
B-OMe-NPs	4T1	PDT; PTT	n/a	735	1	600	n/a	51	36
BQDs-PEG	4T1	PTT	IR thermal camera	808	1	600	n/a	52	37
Y16-Pr-PEG NPs	4T1	PDT; PTT	n/a	808	1	600	n/a	60	38
AuNSPHs	4T1	PTT	IR thermal camera	808	1	300	18.9	52.9	39
PQs-TPA NPs	4T1	PTT	IR thermal camera	808	0.8	600	28	62	40
Mel-pDA-ICG	4T1	PTT	NIR camera	808	1.2	300	n/a	60	41
PT; mPT	4T1	PTT	SolidSpec-3700	808	1.5	600	n/a	n/a	42
M1 NPs	4T1	PTT	Infrared thermography	808	1	600	n/a	55	43
PEGylated Sb-835 NPHs	4T1	PTT	n/a	808	1	540	58	n/a	44
Au_44_MBA_26_-Cy7 NCs	4T1	PTT	n/a	808	1	600	n/a	61.3	45
J-Au-CS-QD	4T1	PTT	IR thermal camera	808	0.5	600	30.4	52.8	46
rGO-AuNP	SKOV-3	PTT	FLIR SC300	1061	0.25	300	n/a	n/a	47
MNFs	4T1	PTT	Fluke Ti400	808	1	600	18.3	51.4	48
W_18_O_49_ nanorods	4T1	CDT; PTT	FLIR A300	808	1	300	n/a	42	49
PD-FA nanoparticles	HeLa	PTT	IR thermal imaging camera (CEM, at an interval of 60 s)	845	1	300	n/a	65	50
DPP-SO; DPP-SS; DPP-SSe NPs	A549	PTT	Fluke Ti480	808	0.8	300	n/a	65	51
Fe-ZDS	EMT-6	PTT	IR thermal imager	808	0.82	360	45	n/a	52
POM	tumor	CDT; PTT	IR thermal images	1060	0.8	600	n/a	n/a	53
DPP-SS; DPP-OF; DPP-SF; DPP-SeF NP	A549	PTT	Fluke Ti480	808	0.75	300	n/a	55	54
MPDA-PEG	A549	PTT	IR camera	808	1; 2	600; 300	26.6	56.7	55
TMB-F4TCNQ; TMB-TCNQ	4T1	PTT	IR imager	1060	1	300	18.8	53.6	56
IDCIC NPs	4T1	PTT	Fluke Ti400	808	1	600	24.5	60.2	57
GBP-FANB	HepG2; PC3	PTT	FLIR Ax5	630	1	600	24.63	54.63	58
IDT-BTzTD Pdots	4T1	PTT	Thermal camera	808	0.8	600		58.5	59
Au@Cu_2_‑xSe NPs	4T1	PTT	Thermal camera	808	1; 1.5	300	31.5	48.9	60
FA-DOX-ICG-PFP@Lip	Y79	PTT	Thermal imaging camera	808	1	300	n/a	50.1	61
MnCO@CNPpy	MCF-7	PTT	Temperature transmitter	808	1	300	n/a	n/a	62
GNS; Silica@GNS	MDA-MB-231	PTT	n/a	808	1	900	n/a	n/a	63
ZPC NPs	4T1	PDT; PTT	Fluke TiS55	660; 808	0.76; 2	300	n/a	53.5	64
GNR@MNPs-Gd-64 Cu	Hep-2	PTT	Fluke Ti400	808	1.5	300	n/a	58.5	65
PFTQ-PEG-Gd NPs	4T1	PTT	FLIR E50	808	1	600	n/a	58.5	66
Ag_2_S@M-P-RGD	HeLa	PTT	IR thermal imaging	808	2	360	n/a	53	67
Au@PB@Cu_2_O@BPQDs/PAH NCs	HeLa	PTT	PTT monitoring system (MG33)	650	1.5	300	16.9	n/a	68

PS: n/a signifies that the information provided is not written or not detailed.
